# From wild shrub to cultivated host: a genetic roadmap for enhancing lac production in *Flemingia semialata*

**DOI:** 10.3389/fpls.2026.1786589

**Published:** 2026-05-07

**Authors:** Jyotirmoy Ghosh, Vaibhav D. Lohot, Nawalesh Kumar Sinha, Vidyapati Vidyakar

**Affiliations:** Agri-Bioresources Augmentation Division, ICAR-National Institute of Secondary Agriculture, Ranchi, Jharkhand, India

**Keywords:** agroforestry roadmap, *Flemingia semialata*, genetic improvement, lac production, orphan crop, phenological asynchrony

## Abstract

The global demand for natural, biodegradable resins such as shellac is increasing, yet production remains severely constrained by reliance on genetically unimproved host plants. *Flemingia semialata* Roxb. has emerged as a superior perennial host for the lac insect (*Kerria lacca*) owing to its rapid growth, adaptability to marginal environments, and high resin yield potential. Despite these advantages, the species remains an underutilized “orphan crop,” lacking systematic genetic characterization and formal breeding programs, thereby limiting its contribution to the lac economy. This review critically examines the biological and genetic constraints impeding the improvement of *F. semialata*, including (i) an uncharacterized and potentially narrow genetic base, (ii) phenological asynchrony with the lac insect life cycle, and (iii) sensitivity to abiotic stresses prevalent in rainfed agroforestry systems. We evaluate the practical applicability of contemporary breeding and genomic tools—ranging from high-throughput phenotyping and transcriptomics to marker-assisted selection and genomics-assisted breeding—for this non-model perennial species. Based on these insights, we propose a structured, three-phase genetic research roadmap to transition *F. semialata* from a wild-collected shrub to a genetically improved host crop. Phase I emphasizes germplasm curation and phenotypic characterization; Phase II focuses on trait genetics and quantitative trait locus discovery; and Phase III targets applied breeding and variety development. This roadmap provides an actionable framework to initiate genetic domestication of *F. semialata*, with significant implications for revitalizing lac-based agroforestry systems, ensuring a stable supply of natural resins, and enhancing livelihood security for millions of smallholder farmers.

## 1 Introduction

Lac, the resinous secretion of the insect *Kerria lacca*, is a vital natural polymer with extensive applications in food, pharmaceutical, and cosmetic industries (Sharma and Jaiswal, 2010). India dominates global shellac production, an industry that supports the livelihoods of 3-4 million tribal and rural families, primarily in eastern and central regions (Yogi et al., 2016). Despite its socio-economic importance, annual lac production (~18,000 tons) achieves less than 25% of its estimated potential (~75,000 tons) (Anonymous, 2023). A primary bottleneck is the dependence on traditional host trees like *Schleichera oleosa*, *Butea monosperma* and *Ziziphus mauritiana*, which have slow growth rates, long gestation periods, and high climate sensitivity.

*Flemingia semialata*, a fast-growing, perennial shrub, offers a transformative alternative. It exhibits superior agronomic traits, including rapid biomass production, efficient nitrogen fixation, and the ability to thrive on degraded soils (Prasad and Jaiswal, 2012). Critically, it demonstrates high compatibility with *K. lacca*, supporting robust insect settlement and potentially higher resin yields (Rahman et al., 2021). Paradoxically, *F. semialata* contributes only 2.5–3.5% to national lac production, a disconnect stemming from its status as a genetically unimproved, wild species (Anonymous, 2020).

Unlike major crops, *F. semialata* exists in a pre-domestication state. There are no defined cultivars, characterized breeding lines, or understanding of its population genetics. Key yield-limiting traits-such as the timing of succulent shoot emergence (phenology) to match insect broods, phloem sap quality, and resilience to drought-remain unstudied from a genetic perspective. This lack of a foundational genetic platform renders conventional and advanced breeding approaches ineffective.

A recent high-quality genome assembly of *Flemingia macrophylla* has revealed that *Flemingia* is most closely related to *Cajanus cajan* (pigeonpea) among sequenced taxa, with an estimated divergence time of 13.2–20.0 million years ago (Ding et al., 2025). Both genera share two whole-genome duplication (WGD) events, indicating conserved synteny and genomic architecture. This close evolutionary relationship positions pigeonpea as an ideal genomic model for *F. semialata* and validates the use of pigeonpea-derived markers, probes, and breeding insights.

This review posits that unlocking the potential of *F. semialata* requires a deliberate transition from a wild shrub to a genetically cultivated host - a challenge emblematic of many orphan crops that remain underserved by modern breeding (Tadele, 2019). We aim to: (1) synthesize the unique genetic and physiological bottlenecks specific to *F. semialata* as a lac host; (2) critically evaluate the applicability of modern plant breeding and biotechnological tools for this orphan crop; and (3) propose a pragmatic, phased research roadmap to establish its first formal genetic improvement program. By providing this focused framework, we seek to catalyze targeted research efforts to develop resilient, high-yielding varieties, thereby securing a sustainable future for lac-based agroforestry systems.

## 2 Botanical features of *Flemingia semialata* and its association with lac insect

### 2.1 Origin

Flemingia genus is distributed in the old-world tropics (Mabberley, 2017) and consists today of 44 species and two varieties (46 taxa). It is thought to have originated in the Indo-Burmese region (Mukerjee, 1953).

### 2.2 Distribution

Bangladesh, Cambodia, China (Guangdong, Yunnan), India (Andhra Pradesh, Assam, Chhattisgarh, Jharkhand, Karnataka, Madhya Pradesh, Maharashtra, Manipur, Meghalaya, Mizoram, Orissa, Uttar Pradesh, Sikkim, Tamil Nadu, Telangana, Tripura and West Bengal), Indonesia (Java), Jamaica, Laos, Myanmar, Thailand, Vietnam.

### 2.3 Phenological and reproductive characteristics

The flowering and fruiting take place from January to April. The plant features axillary and terminal racemes, 9–12 cm long, with hairy, gland-dotted, pedicellate flowers. The calyx is 10–11 mm long with five lanceolate teeth, while the corolla is pale yellow with red striations, comprising a rounded standard petal with two auricles, oblong wing petals, and fused keel petals. Androecium consists of ten diadelphous stamens (9 + 1). The ovary is gland-dotted, hairy, and contains two ovules; the style is swollen at the middle. The fruit is a beaked, turgid pod (16–17 × 7–7.5 mm) containing two brown, mottled, rounded seeds. The hilum is granular and centrally positioned (Lohot et al., 2023).

*Flemingia semialata* Roxb. is an important perennial shrub belonging to the family Fabaceae and serves as a major host plant for the lac insect *Kerria lacca*. The species is widely cultivated in lac-growing regions of India due to its suitability for sustaining lac insect populations and its capacity to produce tender shoots required for insect settlement. Despite its significance in lac production systems, the plant is relatively less familiar to a broader scientific audience outside lac research, making a visual representation of the species valuable for understanding its morphological characteristics and host suitability.

The plant typically grows as a multi-stemmed shrub with profuse shooting and a dense canopy. Young shoots emerge following pruning, providing suitable sites for the settlement of lac insect crawlers. The leaves are trifoliate, and the plant produces racemose inflorescences bearing small papilionaceous flowers typical of legumes. Flowering is followed by pod formation, with each pod containing seeds that contribute to natural regeneration and germplasm maintenance.

Several plant morphological features are relevant to lac cultivation and genetic improvement. These include shoot vigor, branching pattern, and the production of succulent young shoots that support lac insect feeding and development. The phenological synchronization between shoot growth and the life cycle of *Kerria lacca* is particularly important for successful lac establishment and resin production. Furthermore, the plant’s ability to tolerate repeated pruning and its adaptability to marginal environments make it a preferred host species in lac-based agroforestry systems.

**Figure 1:**
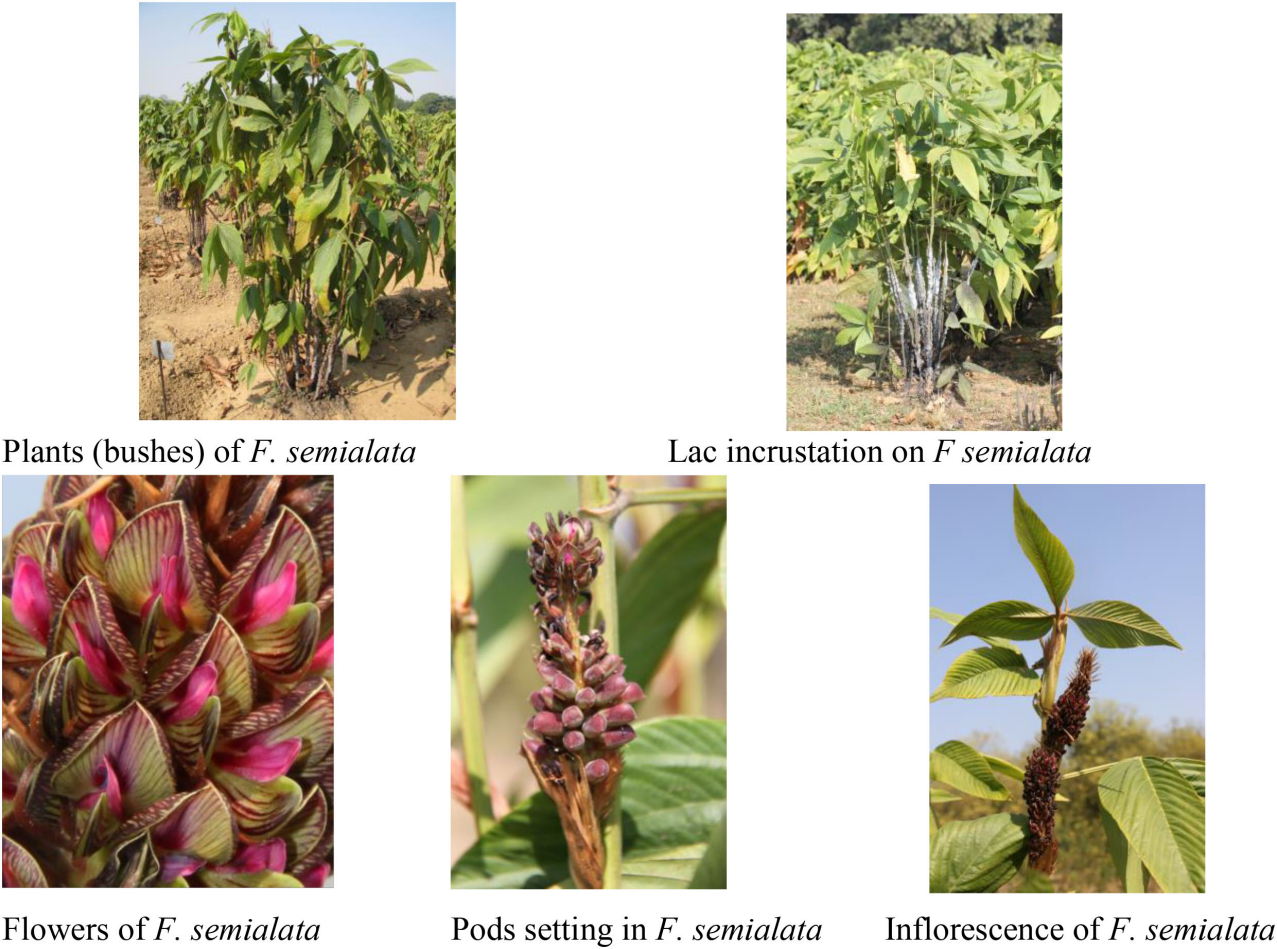
Photographic plate illustrating the botanical features of *Flemingia semialata* and its association with the lac insect (*Kerria lacca*). The plate includes images of the host plant, plant habit (bushes), lac incrustation on the branches, flowers, inflorescence and pod setting in *F. semialata*.

To familiarize readers with the species and illustrate its morphological attributes and association with the lac insect, a photographic plate is presented in **Figure 1**. The plate includes representative images of the plant habit, inflorescence, buds and flowers, pod formation, and lac incrustation on the host branches. *Flemingia semialata* germplasm was brought from International Crops Research Institute for the Semi-Arid Tropics (ICRISAT), Hyderabad, India with the accession number ICPW201 Since then, the germplasm was maintained at the Lac Host-Plant Field Gene Bank at Institute Research Farm, ICAR-NISA, NAMKUM, Ranchi (Jharkhand), India.

### 2.4 Life cycle of Kerria lacca

**Figure 2:**
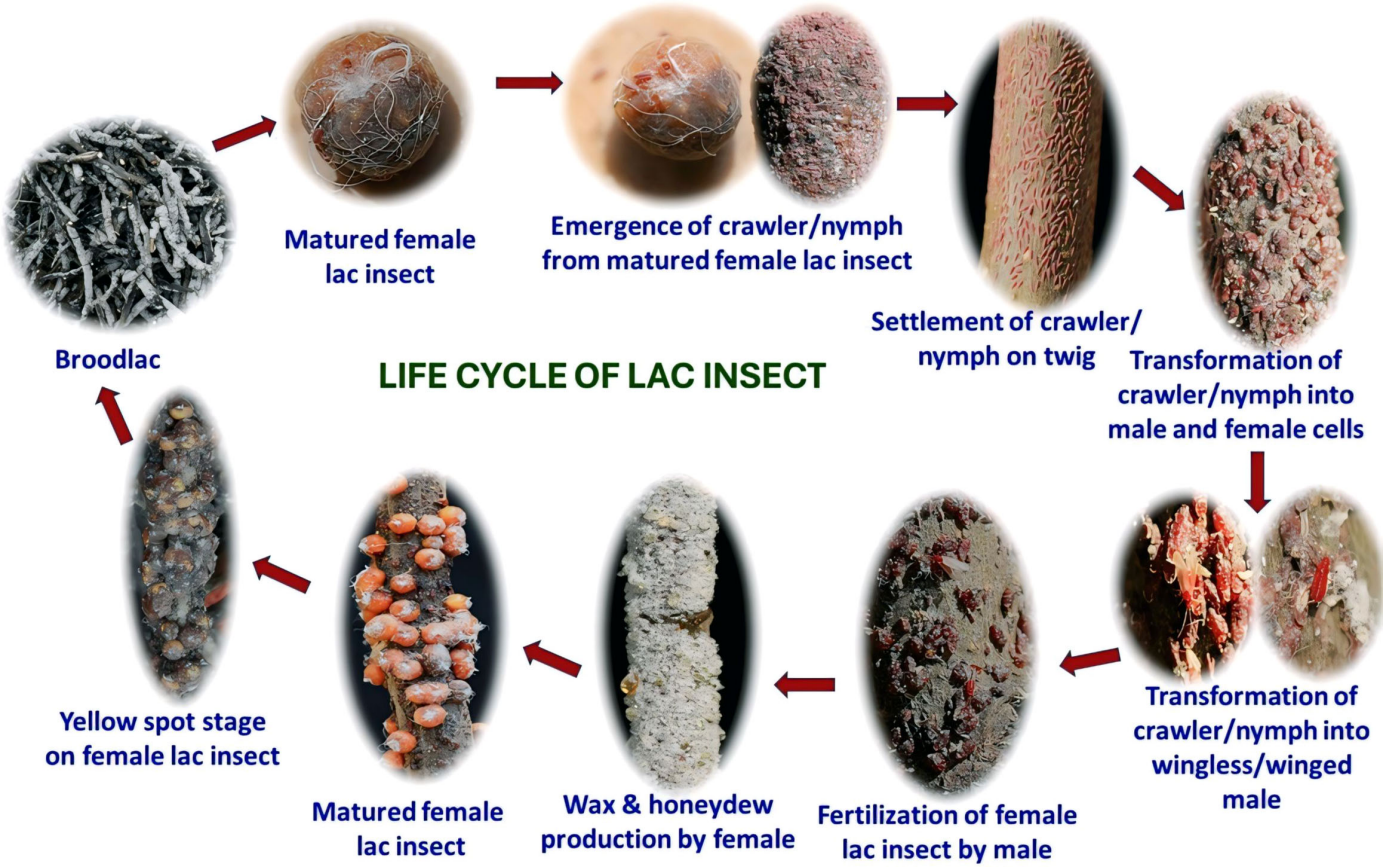
Life cycle of *Kerria lacca.*.

The lac insect *Kerria lacca* is a phloem-feeding scale insect responsible for the secretion of natural resin known as shellac. Its life cycle consists of broodlac, crawler/nymph (first instar), settlement stage, sex differentiation stage (male and female formation), resin, wax & honeydew secretion stage, female lac insect maturation & yellow spot formation stage and finally broodlac formation stage from where crawler/nymph emerges (**Figure 2**).

The crawler stage is the only mobile phase, during which the insect actively seeks suitable host tissue. Successful settlement occurs only on young, tender, and actively grown shoots. Once settled, the insect inserts its stylet into the phloem and begins feeding, gradually secreting resin that forms a protective covering around its body.

## 3 Agronomic importance and genetic potential of *Flemingia semialata* as a lac host

*Beyond its botanical characteristics*, Flemingia semialata *has gained increasing attention as a promising host plant for lac cultivation due to its favorable agronomic and physiological traits. Compared with traditional lac hosts such as* Ziziphus mauritiana, Butea monosperma*, and* Schleichera oleosa*, the specie offers several advantages that make it particularly suitable for intensive lac-based production systems.*

One of the most notable attributes of *F. semialata* is its rapid establishment and early shoot production. The shrub produces harvestable shoots within 10–12 months after planting, enabling early inoculation with lac insects and faster economic returns. In contrast, traditional host trees require a considerably longer establishment period, typically 5–7 years in *Ziziphus mauritiana*, 7–10 years in *Butea monosperma*, and 10–12 years in *Schleichera oleosa* before they become suitable for lac cultivation.

The plant also possesses strong perennial growth and ratooning ability. As a multi-branched shrub, it tolerates repeated pruning and can produce multiple cycles of tender shoots required for lac insect settlement. This characteristic allows farmers to harvest several lac broods from the same plant over successive years through appropriate pruning management, making the system both sustainable and economically attractive.

Another important advantage is its host compatibility with the lac insect (*Kerria lacca*). Preliminary biochemical studies suggest that the phloem sap of *F. semialata* contains relatively higher concentrations of essential amino acids required for lac insect development and resin biosynthesis (Kaushik et al., 2020). Such nutritional suitability may contribute to improved insect survival, feeding efficiency, and ultimately resin yield.

From an agroecological perspective, *F. semialata* also integrates well into agroforestry systems. As a leguminous species, it contributes to soil fertility through biological nitrogen fixation. Its moderate height and shrub architecture enable it to function effectively as an understorey or hedge component in multi-strata cropping systems, supporting diversified production systems in lac-growing regions (Raihan, 2023).

Despite these advantages, the current utilization of *F. semialata* remains largely phenotypic and unstructured. Farmers typically propagate plants from unselected local material, resulting in considerable variability in growth, shoot production, and lac yield. The absence of systematic genetic improvement and germplasm evaluation limits the realization of its full production potential. Therefore, identifying superior genotypes, understanding trait variability, and incorporating these traits into targeted breeding programs are essential steps to transform the species’ anecdotal advantages into measurable genetic gain and consistent productivity.

## 4 The host plant, lac insect and their interaction

The lac insect (*Kerria lacca*) is a unique sedentary phloem-feeding insect cultivated commercially for the production of natural resin (lac), which plays a significant role in supporting forest-based rural livelihoods. Unlike other phloem feeders such as aphids and whiteflies, the lac insect exhibits distinctive biological characteristics, including permanent immobility after settlement, a prolonged life cycle of approximately 4–8 months, and continuous feeding on stem phloem sap throughout its developmental stages. The period extending from post-fertilization (after mating) to insect maturation represents the most metabolically demanding phase of the lac insect life cycle. During this stage, female insects simultaneously sustain embryonic development while actively secreting resin, resulting in a substantial increase in nutrient demand. Consequently, intense phloem sap extraction occurs, coinciding with marked depletion of sugars, proteins, and phenolic compounds in host plant tissues.

The interaction between the lac insect and its host plant represents a finely balanced co-evolutionary relationship. Rather than inducing strong defensive resistance, the host plant predominantly activates tolerance-based physiological responses that allow sustained insect feeding without severe host mortality. Simultaneously, the insect modulates host metabolic pathways to ensure continuous nutritional supply. This adaptive equilibrium enables the survival of both partners and facilitates sustainable resin production. Among all developmental stages, the post-fertilization to maturation phase emerges as a critical window for physiological regulation and management interventions.

Host plant health management should be prioritized during the critical post-fertilization stage.

Selection of cultivation sites must consider host plant phenology, growth habit, and seasonal physiology. Biochemical interactions in lac insects differ fundamentally from those observed in other phloem-feeding insects. Future research should focus on molecular mechanisms underlying host manipulation, genetic variability of both host and insect populations, and determinants of resin production efficiency.

Thus, the lac insect–host plant association exemplifies a specialized plant–insect interaction in which prolonged sedentary phloem feeding induces extensive metabolic reprogramming within the host. This dynamic interaction supports mutual survival while enabling economically important resin production, highlighting its ecological significance as well as its value for sustainable lac cultivation systems (Lohot and Ghosh, 2018; Lohot et al., 2023).

## 5 Core breeding challenges: defining the genetic bottlenecks

The development of improved *F. semialata* varieties is predicated on overcoming specific, genetically tractable constraints.

### 5.1 The genetic diversity black box in *Flemingia semialata*

The characterization of *Flemingia semialata* as a “genetic diversity black box” reflects the striking absence of molecular and genomic information on intraspecific variation, despite its ecological and economic importance as a premier lac host. While its taxonomic identity, geographic distribution, and general growth habit are documented (POWO, 2023), systematic assessments of genetic diversity within and among natural populations are virtually nonexistent. Most existing studies have focused on morphology, agronomic performance, or host suitability for lac cultivation, with minimal integration of molecular tools (Kumar et al., 2017).

Although phylogenetic analyses using nuclear ITS and chloroplast markers have clarified genus-level relationships within *Flemingia* (Wu et al., 2013; Zhang et al., 2015; Egan et al., 2016), *F. semialata* itself remains severely under-represented in molecular datasets. Consequently, species-level patterns of genetic diversity, population structure, and adaptive differentiation remain unresolved.

This knowledge gap has profound implications for crop improvement. Genetic diversity characterization forms the foundation of any effective breeding program; however, for *F. semialata*, this prerequisite is largely absent. Germplasm collections are fragmented, non-systematic, and lack representation across ecological gradients. As a result, information on allelic richness, population structure, and linkage disequilibrium for economically important traits is unavailable, severely constraining parent selection, hybridization strategies, and the application of modern genomics-assisted breeding approaches.

### 5.2 Phenological asynchrony: the prime target

**Figure 3:**
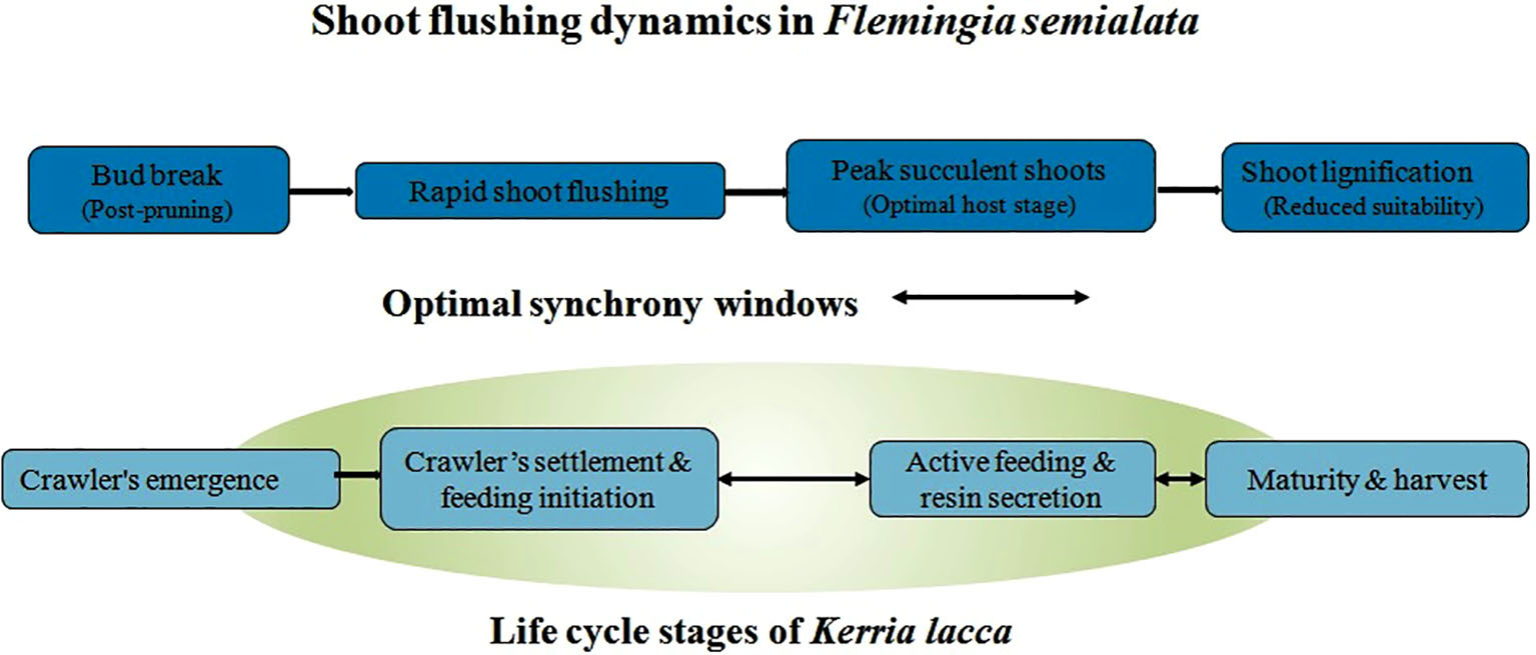
Phenological synchrony between shoot flushing in *Flemingia semialata* and the life cycle of the lac insect (*Kerria lacca*).

Phenological synchrony between host plant shoot emergence and lac insect settlement is a fundamental determinant of lac productivity. *Kerria lacca* requires young, actively growing, and succulent shoots for successful settlement, feeding, and resin secretion (**Figure 3**). However, in *Flemingia semialata*, vegetative flushing is often erratic, prolonged, or poorly synchronized with the fixed inoculation windows of lac strains, leading to sub-optimal insect establishment and uneven colony development (Deb and Chaturvedi, 2010; Meena et al., 2014). Such mismatches frequently result in partial mortality of crawler stages, reduced resin deposition, and high variability in yield across seasons and locations.

Phenological asynchrony is increasingly recognized as a complex quantitative trait governed by the interaction of photoperiod, temperature, soil moisture, and endogenous hormonal signaling pathways, particularly those involving gibberellins, cytokinins, and auxins (Way and Montgomery, 2015; Singh et al., 2021). In perennial leguminous shrubs like *F. semialata*, environmental cues regulating bud dormancy release and shoot initiation appear highly plastic, making phenology unpredictable under rainfed and climate-variable conditions. Climate change–induced shifts in temperature and growing season onset are already documented to advance flowering and leafing in temperate perennials by over 10 days in recent decades (Chmielewski et al., 2018), suggesting similar phenological disruptions in tropical lac systems. Such trends further exacerbate asynchrony, intensifying the mismatch between host readiness and insect life cycle events (Cleland et al., 2007; Parmesan and Hanley, 2015). These biological and phenological constraints directly translate into genetic bottlenecks, which are discussed in the following section.

Successful lac establishment and resin yield depend on the temporal overlap between the emergence of young, succulent shoots following pruning and the crawler settlement phase of the insect. The highlighted optimal synchrony window represents the period of maximum host suitability and insect performance. Phenological asynchrony leads to poor crawler survival and reduced resin secretion, identifying synchronized shoot flushing as a primary breeding target for improving lac productivity and yield stability.

From a breeding perspective, predictable and synchronized shoot flushing emerges as the single most critical trait for stabilizing lac yield. Selection for genotypes exhibiting uniform bud break, rapid post-pruning regrowth, and sustained production of suitable shoots during inoculation periods could dramatically enhance insect settlement success. Comparable phenological selection strategies have proven effective in perennial crops such as tea, coffee, and mulberry, where synchrony with harvesting or insect feeding stages is essential (Banerjee and Mukherjee, 2012; Sarkar et al., 2018). In *F. semialata*, integrating phenological phenotyping with molecular approaches to identify quantitative trait loci (QTLs) and regulatory genes controlling flushing behavior should be prioritized as a cornerstone of host improvement programs.

### 5.3 Abiotic stress sensitivity

Lac cultivation systems are predominantly located on marginal, rainfed lands characterized by shallow soils, erratic rainfall, and poor drainage. Under such conditions, F. semialata frequently experiences both drought and episodic waterlogging stress, each of which significantly impairs shoot vigor, cambial activity, and phloem sap flow- key determinants of host suitability for lac insects (Meena and Choudhary, 2023; Yadav et al., 2020). Drought stresses reduces leaf area, photosynthetic efficiency, and assimilate availability, while waterlogging induces hypoxia in the root zone, disrupting nutrient uptake and hormonal balance (Parent et al., 2008).

These abiotic stresses directly compromise the plant’s capacity to sustain dense lac insect populations, leading to poor settlement, stunted insect growth, and reduced resin secretion. Physiological studies in lac host plants have shown that water stress alters phloem sap composition and reduces carbohydrate translocation, thereby diminishing insect nutrition (Lohot et al., 2019; Meena et al., 2023). Moreover, stress-induced leaf senescence and shoot dieback further narrow the window of suitable feeding sites.

Breeding for abiotic stress resilience in *F. semialata* requires the genetic dissection of key adaptive traits such as deep and plastic root architecture, efficient stomatal regulation, high water-use efficiency (WUE), osmotic adjustment capacity, and, under waterlogging conditions, the formation of aerenchyma and adventitious roots (Fukai and Cooper, 1995; Voesenek and Bailey-Serres, 2015). Advances in tree and shrub genomics suggest that integrating physiological screening with marker-assisted selection or genomic selection could accelerate the development of stress-tolerant lac host ideotypes. Given the increasing climatic uncertainty in lac-growing regions, abiotic stress tolerance should be treated as a co-equal breeding objective alongside phenological synchrony.

### 5.4 The host-insect biochemical interface as a breeding objective

Lac yield is ultimately a function of lac insect health, survival, and resin secretion capacity, all of which are intricately linked to the biochemical composition of host phloem sap. The nutritional quality of the sap-particularly its sugar profile, amino acid composition, and mineral content—directly influences insect metabolism, fecundity, and resin biosynthesis (Kaushik et al., 2020). Furthermore, the physical and chemical dynamics of phloem feeding—including sieve element occlusion, sap viscosity, and nutrient accessibility—are critical determinants of insect feeding efficiency and host suitability (Will and van Bel, 2006). Recent advances in metabolic engineering and plant immunity offer promising routes to tailor phloem chemistry for improved insect performance and crop resistance (Douglas, 2018). An ideal host is therefore characterized not merely by vigorous growth, but by a phloem sap profile optimized for lac insect nutritional requirements.

**Figure 4:**
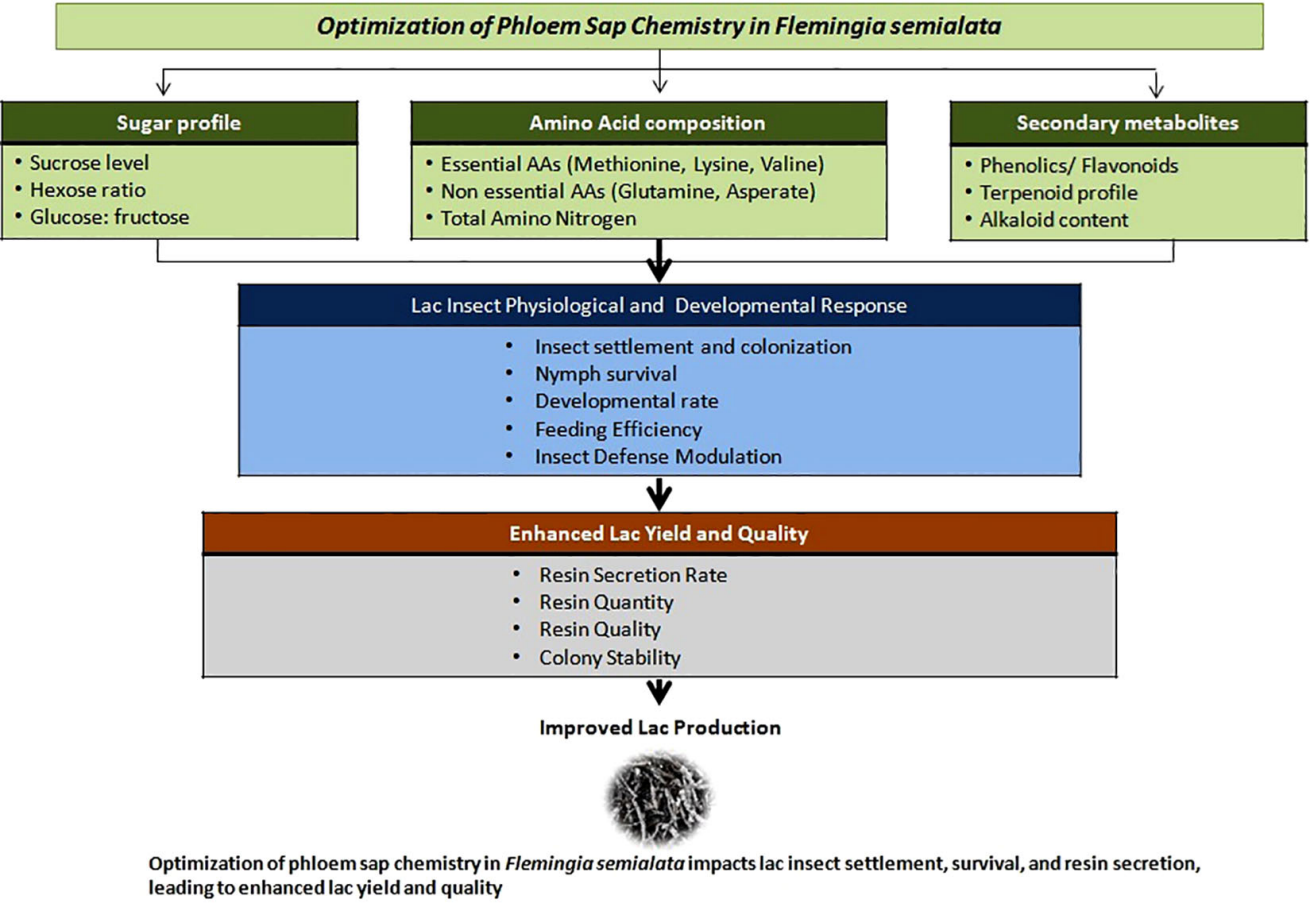
Optimization of phloem sap chemistry in *Flemingia semialata* impacts lac insect settlement, survival and resin secretion, leading to enhanced lac yield and quality.

Empirical studies on lac host plants have demonstrated that higher concentrations of soluble sugars and essential amino acids (such as lysine, methionine, and valine) correlate positively with lac insect growth and resin yield (Ramani et al., 2007; Lohot et al., 2019). Mineral nutrients, particularly potassium, calcium, and magnesium, play critical roles in enzyme activation and resin polymerization processes. Conversely, imbalances in sap chemistry can limit insect feeding efficiency and reduce resin deposition even under otherwise favorable conditions (**Figure 4**).

In addition to primary metabolites, secondary metabolites—such as phenolics, flavonoids, and terpenoids—may influence insect adhesion, settlement behavior, and resistance to natural enemies and microbial pathogens (War et al., 2012). While excessive phenolics may deter feeding, optimal concentrations could enhance plant defense without compromising insect performance, highlighting the need for a finely balanced biochemical profile. This trait has never been genetically targeted in lac host improvement.

Despite its importance, the genetic regulation of phloem sap composition in *F. semialata* remains largely unexplored. Deciphering the genetic architecture underlying “phloem chemistry” through metabolomics, transcriptomics, and quantitative genetics represents a frontier area in lac host improvement. Defining an ideotype-specific sap profile (**Figure 2**) and incorporating it as a selectable breeding trait would mark a paradigm shift from conventional host selection based solely on morphology and growth, toward a more mechanistic, insect-centric breeding strategy.

## 6 Applicable breeding techniques for an orphan crop

It requires a strategic, sequential approach tailored to a species with no prior genomic resources.

The genetic improvement of *Flemingia semialata* demands a carefully phased strategy that acknowledges its status as a non-model perennial species with virtually no existing genomic resources. Unlike major crops, where dense marker platforms and reference genomes are readily available, breeding in *F. semialata* must begin with foundational resource development before advanced tools can be deployed. A strategic, sequential framework- integrating germplasm curation, high-resolution phenotyping, and cost-effective genomic approaches- is therefore essential to unlock its breeding potential.

### 6.1 Pre-breeding strategies for *Flemingia semialata*

Pre-breeding serves as a critical bridge between genetic resource conservation and applied breeding, particularly for under-domesticated species such as Flemingia semialata. It involves the systematic identification, introgression, and stabilization of desirable genes from wild relatives, landraces, and exotic germplasm into intermediate breeding materials, thereby broadening the genetic base of improved populations (Jain and Omprakash, 2019). Globally, crop productivity is increasingly constrained by narrow genetic diversity and the underutilization of available genetic resources, whereas crop wild relatives often harbor valuable alleles conferring resistance to pests and diseases, tolerance to drought and temperature extremes, and enhanced physiological efficiency. However, the direct use of such materials is frequently limited by cross-incompatibility barriers and linkage drag, which pre-breeding seeks to overcome through strategic introgression, backcrossing, and population development (Begna et al., 2023).

For *F. semialata*, pre-breeding assumes particular significance due to its pivotal role as a lac host plant and its relatively narrow genetic base, which increases vulnerability to abiotic stresses and biotic pressures. Key pre-breeding activities for this species include systematic characterization of landraces and wild populations, development of broad-based base populations, exploration of polyploidy induction, and the use of wide and interspecific crosses to generate novel variation (Abebe and Tafa, 2021). Advances in next-generation sequencing, molecular marker technologies, *in vitro* culture, cryopreservation, and genebank management have substantially enhanced the identification, conservation, and utilization of genetic diversity, enabling the discovery of quantitative trait loci (QTLs) and novel genes associated with stress tolerance, growth vigor, and phloem sap quality (Salgotra and Chauhan, 2023). These genetic resources can subsequently be incorporated into breeding pipelines through marker-assisted selection and targeted backcrossing.

Wide hybridization represents a particularly promising pre-breeding strategy for F. semialata. Crosses with closely related species within the Flemingia genus can facilitate the introgression of alleles for improved shoot vigor, pest resistance, and enhanced phloem sap production, while more distant interspecific crosses, although technically challenging, offer opportunities to introduce entirely new adaptive traits. Interspecific hybridization has the potential to transfer resilience to drought, heat, and soil constraints from wild relatives, thereby improving host robustness and yield stability under marginal and rainfed conditions. The resulting hybrid derivatives may exhibit increased stress tolerance, enhanced biomass and sap productivity, improved resistance to pests and diseases, and broader ecological adaptability, collectively expanding the geographical and environmental envelope for sustainable lac cultivation.

The close phylogenetic relationship between Flemingia and Cajanus with an estimated divergence of only 13–20 million years and shared whole-genome duplication events (Ding et al., 2025) provides a strong rationale for leveraging the extensive genomic resources developed in pigeonpea. Pigeonpea has graduated from an ‘orphan crop’ to a ‘genomic resources-rich crop,’ with a draft genome, dense genetic maps, transcriptome assemblies, and validated QTLs for agronomic traits (Varshney et al., 2013; Bohra et al., 2017). These resources can serve as cross-species scaffolds for marker development, candidate gene identification, and comparative mapping in *F. semialata*. This absence of structured diversity analysis not only limits parent selection but also risks genetic vulnerability under climate change, potentially destabilizing lac-based production systems.

Furthermore, successful introgression of traits from tertiary gene pool wild relatives-such as *C. platycarpus*- into cultivated pigeonpea through embryo rescue and advanced backcrossing (Sharma et al., 2020) provides a methodological template for similar wide hybridization efforts in *Flemingia*. The pre-breeding populations developed from such crosses, containing introgression lines with enhanced early maturity, high protein content, and photo-insensitivity (Sharma et al., 2020), demonstrate the feasibility of tapping tertiary gene pools for agronomic improvement.

### 6.2 Marker-assisted selection - a critical appraisal for *Flemingia semialata*

#### 6.2.1 Feasibility and limitations for a perennial shrub

**Marker-assisted selection (MAS)** - the manipulation of target traits through linked DNA markers (Collard and Mackill, 2008) - has accelerated breeding in major crops by enabling early, environment-independent selection (Jiang, 2013). For *Flemingia semialata*, translating MAS from concept to practice requires critical evaluation of its feasibility within the biological and logistical constraints of a perennial, non-model shrub.

#### 6.2.2 Near-term opportunities

The most pragmatic application of MAS in *F. semialata* lies in drought tolerance breeding. Lac cultivation is confined to rainfed marginal lands where water stress severely constrains shoot vigor, phloem sap flow, and insect survival (Meena and Choudhary, 2023). Critically, drought-resilient *F. semialata* genotypes with superior lac productivity under water-limited conditions have already been identified (Ghosh et al., 2017), providing ideal material for dissecting adaptive traits such as deep root systems and stomatal regulation. Once QTLs governing these traits are mapped and validated, MAS could enable rapid identification of drought-tolerant seedlings, bypassing multi-year field evaluations.

#### 6.2.3 Current readiness and constraints

Effective MAS deployment hinges on resources only now being developed. DNA markers—microsatellites or SNPs—must be abundant, genome-wide, and sufficiently polymorphic. Recent progress in DNA extraction standardization and preliminary microsatellite characterization (Kumar and Kumari, 2023) is a critical first step, but current marker density remains insufficient for genome-wide scans, and marker-trait validation in independent backgrounds is lacking.

##### 6.2.3.1 Several factors unique to *F. semialata* may impede MAS efficacy

Genetic background effects: Even robust QTLs may not transfer predictably across populations in this genetically uncharacterized species (Holland, 2004).

QTL reliability: Drought tolerance and phenological synchrony are polygenic; mapping often detects only large-effect regions explaining limited variance (Collard et al., 2005). Imprecise markers risk recombination and selection errors.

Perennial cycle constraints: With 2–4 years needed to evaluate mature traits, the feedback loop between marker discovery and validation is lengthy, increasing costs.

Infrastructure gaps: MAS requires cost-effective genotyping platforms and close collaboration between molecular biologists and breeders- resources limited in lac-growing regions (Jiang, 2013).

##### 6.2.3.2 A realistic path forward

Given these constraints, MAS in *F. semialata* should be a medium-term goal. Priority must be to develop a saturated marker platform (via RNA-Seq or GBS), establish well-phenotyped mapping populations, and validate marker-trait associations across environments. Once robust markers are available, MAS can be integrated for early generation selection of drought-tolerant seedlings, accelerated backcrossing of traits from wild relatives, and maintenance of valuable alleles during population improvement.

Inclusive MAS holds promise for accelerating genetic gain in *F. semialata*, particularly for drought tolerance where genotypic variation is already documented. However, practical deployment requires systematic marker development, rigorous QTL validation, and institutional investment in genotyping capacity. A phased approach- from marker discovery to validation to applied selection- can transition MAS from theoretical possibility to tangible asset in domesticating this underutilized lac host.

### 6.3 CRISPR/Cas9 – a critical appraisal for *Flemingia semialata*

#### 6.3.1 Genome editing (CRISPR/Cas9): prospects and constraints for a perennial shrub

CRISPR/Cas9-mediated genome editing enables precise modification of genes governing traits such as phloem-specific transport and stress responses (Jaganathan et al., 2018), with high-fidelity Cas9 variants now available to minimize off-target effects (Chao et al., 2021). Owing to these recent efforts, the CRISPR/Cas9 system is becoming a revolutionary and flexible tool for genome engineering (Ding et al., 2016). For *Flemingia semialata*, however, the immediate utility of CRISPR lies not in direct variety development but in functional validation of candidate genes emerging from transcriptomic or QTL discovery phases.

At present, CRISPR/Cas9 is best viewed as a functional genomics tool rather than a near-term breeding technology for *F. semialata*. Its most pragmatic application would be to validate the role of genes implicated in key traits—for example, a putative regulator of shoot development identified through RNA-Seq contrasts between synchronous and asynchronous flushing genotypes, or a transporter gene associated with phloem loading efficiency. Such validation can confirm gene function before investing in marker development or selection programs.

#### 6.3.2 Several factors currently limit CRISPR deployment in *F. semialata*

Genetic transformation barriers: CRISPR requires efficient transformation and regeneration protocols, which are not yet established for this species. Most perennial woody legumes remain recalcitrant to stable genetic transformation, making routine editing impractical.

Genomic resources: Effective guide RNA (gRNA) design requires high-quality genomic sequence information to identify target sites and predict off-target effects. While tools such as CRISPR-P 2.0 (Liu et al., 2017) and CRISPR-Local (Sun et al., 2019) facilitate gRNA design in plants, their application in *F. semialata* is constrained by the absence of a reference genome.

Perennial lifecycle: Even if transformation were achieved, the extended juvenile phase of *F. semialata* (2–3 years to maturity) would delay phenotypic evaluation of edited lines, slowing the validation feedback loop.

Regulatory and public perception considerations: As a non-food industrial crop, *F. semialata* may face fewer regulatory hurdles than food crops. However, site-directed nuclease (SDN) technologies are still subject to varying regulatory frameworks internationally, which could complicate future deployment.

Given these constraints, CRISPR/Cas9 in *F. semialata* should be pursued as a long-term research tool rather than an immediate breeding solution. Priorities include:

- Establishing efficient transformation and regeneration protocols for *F. semialata*—a prerequisite that may require significant investment and collaboration with model legume systems.
- Leveraging transcriptome assemblies as temporary references for gRNA design until a genome sequence becomes available.
- Focusing initial efforts on loss-of-function validation of strong candidate genes (e.g., flowering time regulators, drought-responsive transcription factors) in rapid-cycling systems or heterologous models where feasible.

CRISPR/Cas9 offers powerful capabilities for gene function validation and, ultimately, trait improvement in *F. semialata*. However, its practical application is contingent upon overcoming fundamental barriers in genetic transformation, genomic resources, and perennial phenotyping timelines. For the near term, CRISPR should be viewed as a complementary tool to dissect gene function, informing rather than replacing conventional and marker-assisted breeding approaches. As foundational resources develop, genome editing may eventually contribute to the genetic domestication of this underutilized lac host.

### 6.4 Targeting induced local lesions in Genomes

The foundational development of high-throughput mutation detection pipelines demonstrated that TILLING can rapidly identify allelic variants with defined genetic changes, facilitating both gene function analysis and the discovery of novel, agronomically useful phenotypes (Colbert et al., 2001). In the context of *F. semialata*, TILLING populations could be exploited to generate and screen mutations in candidate genes involved in phloem sap composition, stress-responsive pathways, and shoot development, providing valuable allelic diversity without the regulatory constraints associated with transgenic approaches.

TILLING, a non-GMO method to identify beneficial mutations in genes controlling sap yield and drought tolerance (Tadele, 2016) may be utilized in *F. semialata*. Recent advancements improve mutation detection efficiency (Kurowska et al., 2011; Chantreau et al., 2013; Suzuki et al., 2008). Chen et al., 2012, Developed and characterize a new TILLING population of common bread wheat (*Triticum aestivum* L.). Aslam et al., 2016 describes the development and application of COTIP, a cotton TILLING platform, for plant improvement and reverse genetic studies. Colbert et al. (2001), describes the development of a method for efficiently detecting induced point mutations in plants. This approach allows researchers to rapidly identify and isolate mutant plants with specific genetic alterations, which is valuable for studying gene function and identifying novel phenotypes. Dalmais et al. (2008) describes UTILLdb, a resource for both forward and reverse genetics in *Pisum sativum* (pea). Jankowicz-Cieslak et al. (2012) focuses on the induction, rapid fixation, and retention of mutations in vegetatively propagated banana plants. Kumar et al. (2013) used SMART – Sunflower Mutant population And Reverse genetic Tool for crop improvement describes the development of a TILLING resource for sunflower, a valuable tool for crop improvement.

### 6.5 Foundational step: germplasm curation and high-throughput phenotyping

The immediate priority for any improvement program in *F. semialata* is the systematic collection, conservation, and characterization of genetically diverse germplasm. Given the species’ wide natural distribution across the Indian subcontinent, Indo-China, and southern China, pan-geographic germplasm collection is necessary to capture adaptive variation shaped by contrasting climatic, edaphic, and biotic environments. Such collections form the biological backbone for all downstream genetic, genomic, and breeding studies.

Equally critical is the development of standardized and reproducible phenotyping protocols for key host traits influencing lac productivity. Priority traits include the timing and synchrony of vegetative flushing, duration of the shoot succulent phase suitable for lac insect settlement, shoot vigor and branching intensity, phloem exudation capacity, and biochemical attributes of phloem sap. In addition, robust indices for abiotic stress responses—particularly drought tolerance and waterlogging resilience—must be incorporated, as lac cultivation is largely confined to marginal, rainfed landscapes. Once sufficient diversity is assembled and phenotyped, the establishment of a core collection representing the maximum genetic and phenotypic variation with minimal redundancy becomes imperative. This curated core set will serve as a permanent reference population for genetic diversity analysis, gene discovery, and association studies, while also functioning as a practical breeding resource. In perennial species, such rationalized germplasm management significantly enhances efficiency and reduces the logistical burden of long-term field evaluation.

## 7 Genomic tools for gene discovery in a non- model species

Beyond QTL mapping and association studies, genomic selection (GS) represents a transformative approach for perennial crop improvement. By using genome-wide marker data to estimate breeding values, GS can accelerate selection cycles and improve genetic gain for complex, polygenic traits such as stress tolerance and phenological synchrony. The successful deployment of GS in fast-growing perennials like Eucalyptus underscores its applicability to genetically underexplored species such as *F. semialata* (Grattapaglia et al., 2018).

Genomics-Assisted Breeding (GAB) in grain legume crops has lagged behind in terms of product delivery; however, genotyping-based selections are now increasingly embraced in breeding programs (Varshney et al., 2021). To drive these programs forward, the detailed reconstruction of grain legume domestication processes provides a roadmap for breeders to integrate beneficial alleles into current breeding pools, ultimately improving crop performance and stress tolerance (Bohra et al., 2022). For gene discovery in non-model species where a reference genome is absent, implementing a cost-effective strategy becomes essential for identifying these critical genomic tools.

The feasibility of GS in *F. semialata* is further supported by progress in its close relative, pigeonpea. Pigeonpea has moved beyond simple MAS to embrace genomic selection (GS) and marker-assisted recurrent selection (MARS) as strategies to capture small-effect QTLs governing complex traits (Varshney et al., 2013). The development of dense genetic maps, QTL maps for drought tolerance-related traits, and genome-wide marker platforms in pigeonpea (Bohra et al., 2017; Varshney et al., 2013) provides a methodological blueprint for GS implementation in *F. semialata*. Moreover, the conserved genome architecture between the two genera (Ding et al., 2025) suggests that prediction models and marker densities optimized in pigeonpea may offer useful parameters for designing GS experiments in *Flemingia*.

### 7.1 Transcriptome sequencing (RNA-seq)

RNA-Seq is a next-generation sequencing (NGS)–based approach that profiles the complete set of RNA transcripts expressed in a tissue or organism at a given developmental stage or environmental condition. Unlike DNA-based methods, RNA-Seq provides a dynamic snapshot of gene activity, enabling the identification of genes directly involved in phenological regulation, stress responses, and host–insect interactions. For *F. semialata*, transcriptome profiling of contrasting tissues—such as flushing versus dormant buds, or stressed versus non-stressed plants—offers a highly efficient route to discover candidate genes underlying priority breeding traits.

Technically, RNA-Seq involves the isolation of high-quality total RNA from target tissues, followed by fragmentation, conversion into complementary DNA (cDNA), and the construction of sequencing libraries. These libraries are sequenced using high-throughput platforms, generating millions of short reads that are assembled *de novo* or mapped to closely related reference genomes. Subsequent bioinformatic analysis enables transcript identification, quantification of gene expression levels, detection of alternative splicing events, and discovery of sequence variants such as single nucleotide polymorphisms (SNPs) (Nagalakshmi et al., 2010; Wang et al., 2009).

Beyond gene discovery, RNA-Seq offers several advantages that make it particularly suitable for non-model perennial species. It does not require prior genomic information, allows the identification of novel genes and regulatory elements, and provides markers directly linked to expressed and potentially functional regions of the genome. Compared with earlier approaches such as microarrays, RNA-Seq offers superior sensitivity, broader dynamic range, and the ability to capture rare transcripts and allele-specific expression (Nagalakshmi et al., 2010; Saeidian et al., 2020).

Nevertheless, challenges remain, particularly in terms of data analysis complexity and the requirement for specialized bioinformatics expertise. High sensitivity may also introduce noise in low-abundance transcripts, necessitating careful experimental design and validation. Despite these limitations, RNA-Seq remains the most informative and cost-effective first step for functional genomics and marker development in *F. semialata* (Deshpande et al., 2023).

For transcriptome analysis in *F. semialata*, the availability of comprehensive pigeonpea transcriptome assemblies (Bohra et al., 2017) provides an invaluable reference for cross-species mapping of RNA-Seq reads. In the absence of a *F. semialata* reference genome, reads can be mapped to the pigeonpea transcriptome to identify conserved genes, alternative splicing events, and sequence variants. This approach has been successfully applied in other orphan legumes and can substantially reduce the bioinformatics burden associated with *de novo* assembly. Additionally, the first set of EST resources developed in pigeonpea for gene discovery and marker development (Bohra et al., 2017) can guide targeted transcriptome sequencing strategies in *F. semialata*, particularly for stress-responsive genes and pathways governing phloem function and phenology.

### 7.2 Reduced-representation genotyping (GBS/RAD-seq)

To complement transcriptomic data and enable genome-wide diversity analysis, reduced-representation genotyping approaches such as Genotyping-by-Sequencing (GBS) or Restriction site–Associated DNA sequencing (RAD-seq) are particularly valuable. These methods generate thousands of genome-wide SNP markers at moderate cost by sequencing reproducible subsets of the genome. Once a sufficiently large and well-phenotyped germplasm panel (typically >200 accessions) is available, GBS or RAD-seq can be used to assess population structure, genetic diversity, and linkage disequilibrium patterns across the species’ range.

Importantly, these marker datasets also lay the foundation for Genome-Wide Association Studies (GWAS), enabling the identification of marker–trait associations for complex traits such as flushing synchrony, shoot vigor, stress tolerance, and phloem sap attributes. In perennial species where controlled crosses are time-consuming, association mapping provides a powerful alternative to classical linkage analysis.

### 7.3 Genetic linkage and QTL mapping

While association studies are informative, controlled biparental populations remain essential for validating trait architecture and estimating genetic effects with higher precision. Strategic crosses between parents exhibiting contrasting phenotypes—such as synchronous versus asynchronous flushing, or stress-tolerant versus stress-sensitive genotypes—should be prioritized. Using SNP markers derived from RNA-Seq or reduced-representation genotyping, a first-generation genetic linkage map can be constructed.

Phenotyping segregating populations such as F_2_ or recombinant inbred lines (RILs) across environments will enable quantitative trait locus (QTL) mapping for key breeding traits, including flush timing, duration of shoot succulence, phloem sap quality, and abiotic stress responses. Even low- to medium-density maps can provide valuable insights into the genetic control of these traits and help identify genomic regions suitable for marker-assisted selection.

### 7.4 Synthesis and breeding implications

Taken together, this integrated framework—from germplasm curation and phenotyping to transcriptomics, reduced-representation genotyping, and QTL analysis—provides a realistic and scalable pathway for modern breeding in *F. semialata*. Rather than attempting to leap directly to whole-genome sequencing, this stepwise strategy maximizes biological insight while remaining economically and technically feasible. Implemented systematically, it can transform *F. semialata* from a genetically underexplored lac host into a scientifically tractable and improvable agroforestry species.

## 8 A phased genetic improvement roadmap

**Figure 5:**
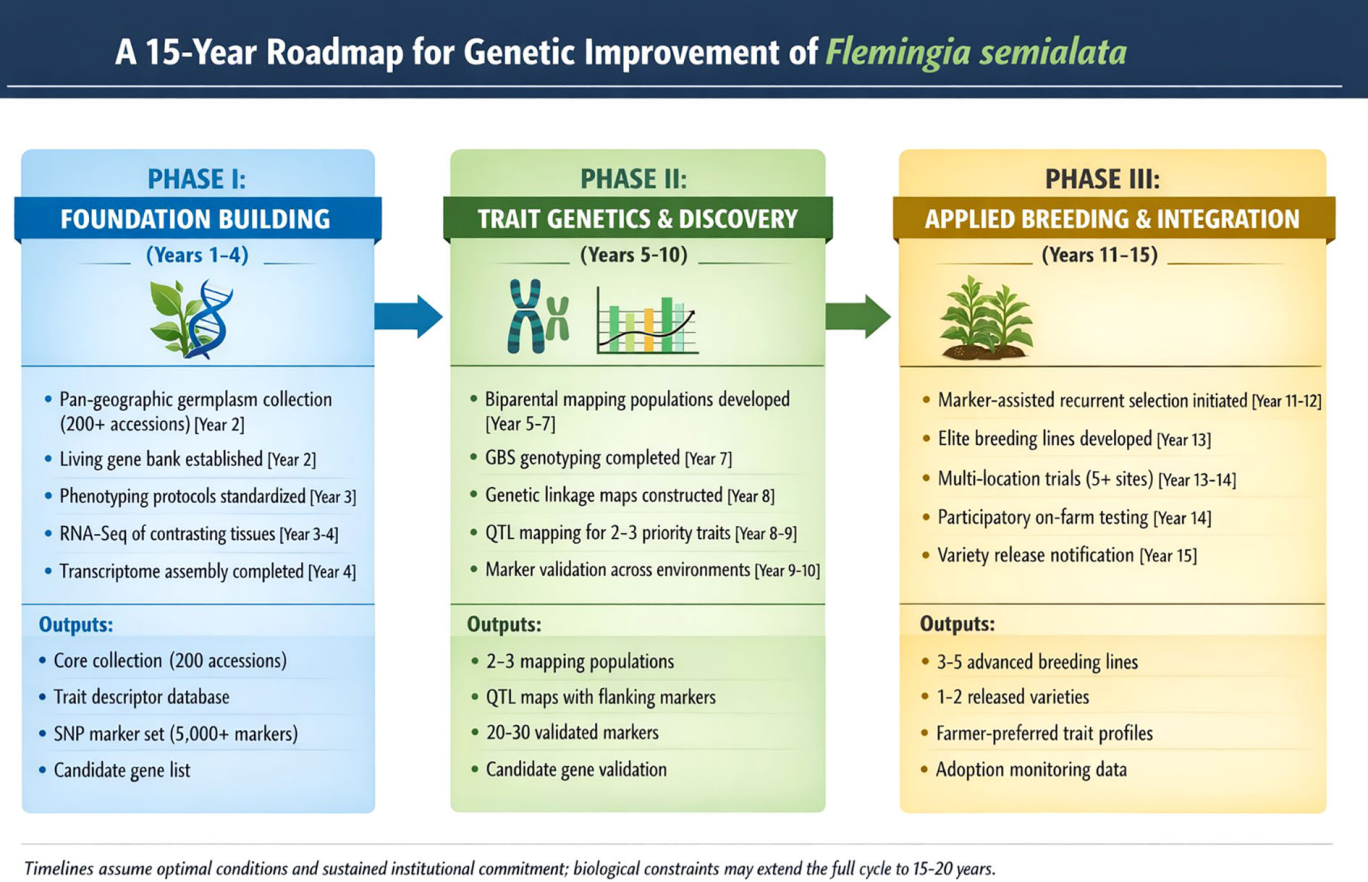
A phased genetic improvement roadmap for the domestication of *F. semialata* as an improved lac host.

To transition *Flemingia semialata* from an underexplored lac host into a genetically improvable agroforestry species, we propose a structured, decade-long, interdisciplinary roadmap comprising three sequential and interlinked phases (**Figure 5**). We propose a *Host–Insect Synchrony Breeding Model (HISBM)* for lac systems. This phased approach is designed to progressively build foundational resources, unravel trait genetics, and ultimately translate scientific gains into deployable breeding outputs.

### 8.1 Phase I: foundation building (years 1–4)

The primary objective of Phase I is to establish the essential genetic, phenotypic, and genomic resources required for systematic improvement. This phase emphasizes comprehensive germplasm exploration across the species’ natural and cultivated range, followed by the establishment of a geo-referenced living gene bank to safeguard and utilize collected diversity. Parallel efforts will focus on developing and standardizing high-throughput phenotyping protocols for priority traits, including flushing synchrony, shoot succulence duration, phloem sap attributes, and abiotic stress responses. To initiate molecular resource development in this non-model species, transcriptome sequencing of key tissues and developmental stages will be undertaken to identify candidate genes and generate SNP markers.

Insights from pigeonpea genetic diversity studies can inform germplasm collection strategies in *F. semialata*. Analysis of wild *Cajanus* species has revealed that wild relatives harbor abundant allelic variation- up to 75% greater polymorphism than cultivated pigeonpea- with a severe domestication bottleneck evident in the cultivated gene pool. This underscores the importance of comprehensive collection of wild *Flemingia* populations before similar genetic erosion occurs.

Furthermore, studies on *C. scarabaeoides*, a wild relative from the secondary gene pool of *C. cajan*, have identified accessions with early flowering (<80 days), high pod set (4–6 seeds/pod), and elevated trichome density conferring pest resistance (Cambridge.org). These findings highlight the types of valuable traits that may exist in *Flemingia* wild relatives and justify systematic germplasm exploration across the genus.

The development of pre-breeding populations utilizing wild *Cajanus* species, such as the introgression lines derived from *C. platycarpus* (tertiary gene pool) that combine high yield with enhanced grain nutrients (Sharma et al., 2020), demonstrates a successful model for tapping wild diversity. Similar approaches-employing embryo rescue where necessary—could be adapted to introgress traits from wild *Flemingia* species into cultivated *F. semialata*.

The expected outcomes of Phase I include a well-characterized core germplasm collection, standardized trait descriptors, robust SNP marker sets, and a publicly accessible transcriptome database that will serve as a foundational platform for subsequent genetic analyses.

### 8.2 Phase II: trait genetics and discovery (years 5–10)

Building on the resources generated in Phase I, Phase II aims to elucidate the genetic architecture of key traits governing host suitability and lac productivity. Population genetic analysis of the core collection using reduced-representation genotyping approaches such as genotyping-by-sequencing (GBS) will provide insights into genetic diversity, population structure, and linkage disequilibrium. In parallel, biparental mapping populations will be developed by crossing contrasting genotypes for major constraint traits, such as synchronous versus asynchronous flushing.

Using molecular markers derived from transcriptomic and GBS datasets, genetic linkage maps will be constructed, enabling quantitative trait locus (QTL) mapping for two to three high-priority traits. This phase is expected to yield the identification of major QTLs and associated markers, improved understanding of population structure, and validation of candidate genes controlling critical phenological and physiological traits.

### 8.3 Phase III: applied breeding and integration (years 11–15)

The final phase focuses on translating genetic knowledge into tangible breeding products and facilitating their adoption. Marker-assisted recurrent selection or targeted hybridization programs will be initiated using validated markers linked to key traits. Elite breeding lines will undergo multi-location evaluation to assess yield stability, adaptability, and performance under diverse agro-ecological conditions. Importantly, participatory on-farm testing will be integrated into this phase to incorporate farmer and stakeholder preferences, ensuring relevance and accelerating adoption. The principal outcome of Phase III will be the release of first-generation, genetically improved *F. semialata* varieties with documented superiority in traits directly linked to lac production, resilience, and system sustainability.

### 8.4 Overall significance

Collectively, this phased roadmap provides a realistic and scalable pathway for integrating modern genetic and genomic tools into *F. semialata* improvement. By aligning resource development, discovery research, and applied breeding within a single strategic framework, the proposed approach bridges the gap between fundamental science and field-level impact, laying the foundation for sustainable enhancement of lac-based agroforestry systems. This framework provides the first structured pathway for genetic domestication of *F. semialata*, with the potential to enhance lac productivity by stabilizing host–insect synchrony under climate variability.

## 9 Conclusion and future perspectives

*F. semialata* stands at a critical juncture. While its agronomic attributes position it as a keystone species for a sustainable lac industry, the absence of genetic improvement confines it to an underperforming and unreliable role. This review emphasizes that realizing its full potential requires not incremental agronomic adjustments, but a deliberate process of genetic domestication.

The proposed phased roadmap offers a realistic and scalable pathway to bridge this gap, beginning with systematic germplasm characterization and advancing toward marker-assisted breeding. Its successful implementation will depend on close interdisciplinary collaboration among geneticists, entomologists, physiologists, and agroforesters.

Lac production in *F. semialata* can be conceptualized as a triadic system involving host genotype, insect biology, and environment, where productivity is maximized at the intersection of phenological synchrony, nutritional suitability, and climatic stability.

Investing in the genetic improvement of *F. semialata* promises benefits extending well beyond enhanced lac yield. It can strengthen the resilience of rainfed agroforestry systems, stabilize incomes for vulnerable farming communities, and secure the supply chain of a valuable natural bio-product. By transitioning *F. semialata* from a wild shrub to an improved lac host, a durable foundation for the future of the lac industry can be established.
